# Barettin, a Nonopioid, Nonhallucinogenic Marine Natural Product with Antihyperalgesic Properties Mediated by 5HT2A Inverse Agonism

**DOI:** 10.1021/acs.jnatprod.6c00169

**Published:** 2026-03-31

**Authors:** Caleb A. Seekins, Michael Okine, Emily G. Forrest, Timothy Chavez, Alexandra Stump, Vishal Kaleeswaran, Jerry E. Carr, Christopher Hulme, Paco Cardenas, Todd W. Vanderah, John M. Streicher, Christopher Cartmell

**Affiliations:** † Department of Pharmacology, College of Medicine, 12216 University of Arizona, Tucson, Arizona 85724, United States; ‡Department of Pharmacology & Toxicology, College of Pharmacy, and §Department of Chemistry and Biochemistry, College of Science, University of Arizona, Tucson, Arizona 85724, United States; ∥ Pharmacognosy, Department of Medicinal Chemistry, 8097 Uppsala University, 752 37 Uppsala, Sweden; ⊥ Museum of Evolution, Uppsala University, 752 37 Uppsala, Sweden; # Comprehensive Center for Pain and Addiction, 8041 University of Arizona, Tucson, Arizona 85724, United States; ∇ University of Arizona Cancer Center, University of Arizona, Tucson, Arizona 85724, United States; ○ The Center for Applied Nano Bioscience and Medicine, College of Medicine, University of Arizona, Phoenix, Arizona 85004, United States

## Abstract

Despite increasing interest, there remain limited treatments for chronic pain, with opioids continuing to be one of the top prescribed medications. Marine natural products present a wealth of untapped potential for new treatments for chronic pain. In this study, we investigated the analgesic effects of the sea sponge ligand barettin in a mouse model of chemotherapy-induced peripheral neuropathy (CIPN). Barettin exhibited efficacious antihyperalgesic activity in male mice with no efficacy shown in females. Through the use of a PRESTO-Tango assay, we found that barettin acts as an inverse agonist at the 5HT2A receptor while also presenting no activity at the μ-opioid receptor. Head twitch experiments confirmed no hallucinogenic activity, suggesting that barettin may be a promising nonopioid, nonhallucinogenic, marine-derived therapeutic agent for the treatment of chronic pain.

## Graphical abstract

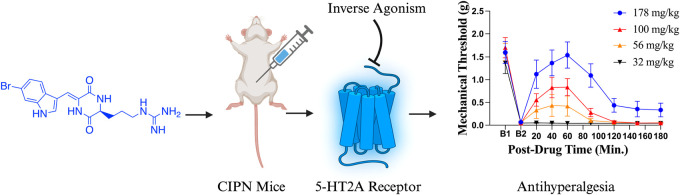

Chronic pain is one of the most common chronic conditions in the United States, affecting 20.5% of adults, and results in a significant economic burden.^1^ In the past three decades, our understanding of pain circuitry and intracellular mechanisms have increased substantially. Yet, despite this new understanding, there has been limited translation of these advancements into novel and effective treatments that overcome the therapeutic limitations of current drugs.^2–4^ Current options for pain management remain limited, with opioids considered the gold standard for treating moderate to severe pain. Although opioids are highly effective, they are associated with profound drawbacks, including tolerance, dependence, addiction, and life-threatening respiratory depression, which have fueled the ongoing opioid crisis.^5–7^ These issues are further compounded in the treatment of chemotherapy-induced peripheral neuropathy (CIPN), a debilitating condition that results from the administration of neurotoxic chemotherapeutic drugs. CIPN is a persistent and severe pain that can lead to lowered doses or discontinuation of chemotherapy agents, increasing patient mortality and potentially leading to retained pain even after the end of treatment.^8^ These challenges underscore the urgent need to identify novel nonopioid alternatives for pain therapeutics. While effective preclinical candidates have been identified, translation into clinical therapy has been limited.^9,10^ Thus, further work is required to identify additional compounds with the aim of identifying one which can be effectively translated into the clinic.

Natural products have long played an essential role in pain management with notable examples including morphine, acetylsalicylic acid, and cannabinoids.^11–14^ While terrestrial natural products have long been investigated, the marine environment remains a mostly unexplored reservoir of chemical diversity. Although oceans cover more than 70% of the Earth’s surface, only a small fraction of this ecosystem has been investigated for its biological and chemical potential. These numbers suggest that marine bioprospecting holds immense promise for the discovery of novel natural product therapeutics.^15–18^ This is further supported by the wide range of pharmacologically active secondary metabolites produced by marine organisms, including fungi, cyanobacteria, cone snails, sponges, soft corals, and tunicates. Many of these marine-derived compounds exhibit unique structures and interact with disease targets in humans through novel mechanisms of action.^19,20^ This includes the treatment of severe, intractable pain, as seen with the analgesic properties of ziconotide, developed from the venom of the cone snail.^21^

Barettin was originally discovered in 1986 from the sponge *Geodia barretti*. Subsequent investigations have uncovered that barettin has pronounced antibiofouling properties, with an ED_50_ value of 0.9 μM for inhibition of barnacle larvae settlement.^22–24^ Barettin features a brominated tryptophan and an arginine that are cyclized in a head-to-tail manner to form a 2,5-diketopiperazine (DKP) motif which enables side chain modifications, rendering barettin a compelling scaffold for future drug development.^25–28^ Despite considerable interest in barettin and earlier reports suggesting affinity for the 5HT2A and 5HT2C receptors, its precise mechanism of action has remained ambiguous.^29^ 5HT receptors have been strongly linked to pain and ligands for these receptors remain a promising target for future analgesics.^30^ For this reason, in this work we investigated barettin’s ability to act as a treatment for chronic pain. Ultimately, we identified barettin as a promising nonopioid, nonhallucinogenic therapeutic candidate for chronic pain. Using a chemotherapy-induced peripheral neuropathy model, we demonstrate its analgesic efficacy and provide evidence that its pharmacological activity is mediated through inverse agonism at the 5HT2A receptor.

## Results and Discussion

### Antihyperalgesic Activity of Barettin

To investigate the antihyperalgesic potential of barettin, we utilized a CIPN model established through repeated administration of paclitaxel in male and female CD-1 mice. Following confirmation of neuropathic pain development via von Frey testing, the mice were randomized into treatment groups to receive various doses of barettin or vehicle. To capture a comprehensive dose–response profile, dosing was performed in quarter-logarithmic increments spanning from 32 to 178 mg/kg via intraperitoneal injection. In male mice, barettin produced a clear dose-dependent attenuation of mechanical allodynia (Figure 1A,B). At the lower dose of 56 mg/kg, a modest increase in paw withdrawal thresholds was first observed, while an intermediate dose (100 mg/kg) resulted in significant antihyperalgesic activity. The highest dose tested (178 mg/kg) elicited a robust and sustained antihyperalgesic effect. Notably, at ∼60 min post administration, the highest dose tested effectively returned thresholds to pre-CIPN levels and approached the efficacy of the known potent analgesic, oxycodone. This suggests that at 178 mg/kg Barettin achieves full efficacy. In contrast, female mice failed to exhibit any antihyperalgesic effect across the same dosing range (Figure 1C,D). ED50 value and control analgesic oxycodone is displayed in Figure 1E,F.

**Figure 1:**
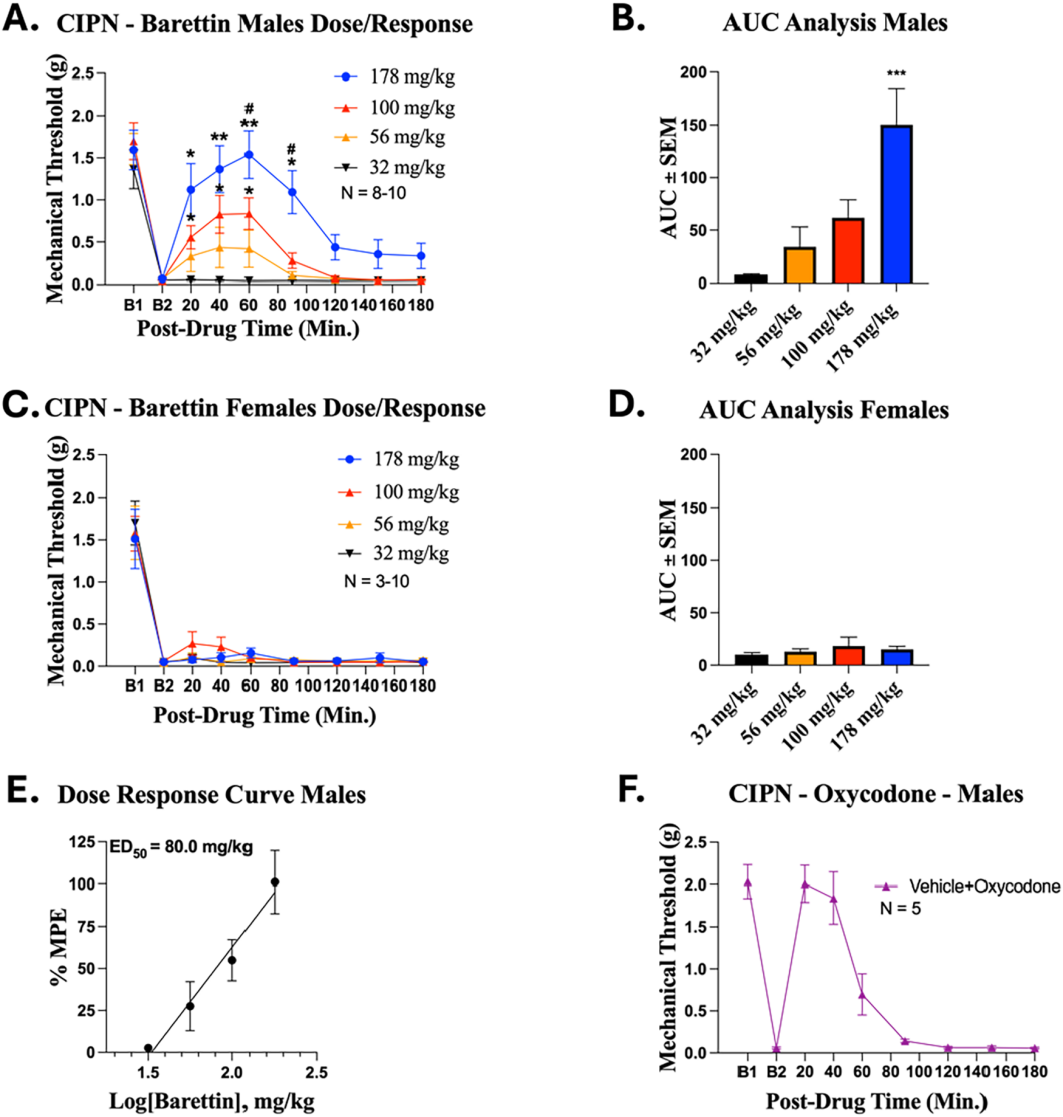
Barettin shows sex-specific antihyperalgesic activity in mice with CIPN. Male and female CD-1 mice had CIPN induced and measured as described in the Methods. Data shown is the mean ± SEM, performed in 1–2 technical replicates for each experiment, with sample sizes noted in each graph. Sample size in female mice, was limited by toxicity at the highest tested dose (*n* = 3) and further testing was not performed due to animal welfare concerns. B1 = baseline prior to CIPN induction, B2 = baseline following CIPN induction. Varying doses of Barettin were injected IP, with mechanical allodynia measured for male (A) and female (C) mice. *, ** = *p* < 0.05, 0.01 vs same time point 32 mg/kg group and ^#^ = *p* < 0.05 vs same time point 56 mg/kg group determined by 2 Way ANOVA with Tukey’s post hoc test. The Area Under the Curve (AUC) for antihyperalgesic effects were analyzed for male (B) and female (D) mice, as described in Methods. *** = *p* < 0.001 vs 32 mg/kg dose, determined by 1 Way ANOVA with Dunnet’s post hoc test. (F) A dose response curve was analyzed for male mice as described in Methods, with ED_50_ denoted. (E) Vehicle (10% DMSO, 10% Tween80, 80% USP Saline) was injected IP 20 min prior to IP injection of 5 mg/kg Oxycodone with mechanical allodynia measured for males as a comparison to (A) and (C). *F* and *P* values are provided in Table S1.

This pronounced divergence between male and female cohorts highlights a sex-dependent pharmacological profile for barettin. This finding is not without precedent, as sex differences in drug-induced analgesia have been widely reported across multiple drug classes.^31^ Thus, the sex-specific effects observed here are consistent with evidence that biological sex strongly influences pain processing and response to analgesics.^32^ Our hypothesized mechanism for this sex difference if further elaborated on in the section titled sex differences in antihyperalgesic efficacy.

### Barettin Acts as an Inverse Agonist at the 5HT2A Receptor

Previous reports have linked barettin to the 5HT2 pathway, though functional activity of barettin has not been investigated.^29^ To assess this, we employed the PRESTO-Tango assay to identify barettin’s functional activity at the 5HT2A receptor. As a positive control, the selective 5HT2A agonist TCB-2 was used to confirm assay activity. TCB-2 elicited robust receptor activation, confirming both the sensitivity of the assay and the functional integrity of the 5HT2A receptor system (Figure 2A). Under identical experimental conditions, barettin demonstrated an inverse agonist profile, reducing basal receptor activity relative to the vehicle control (Figure 2B).

**Figure 2:**
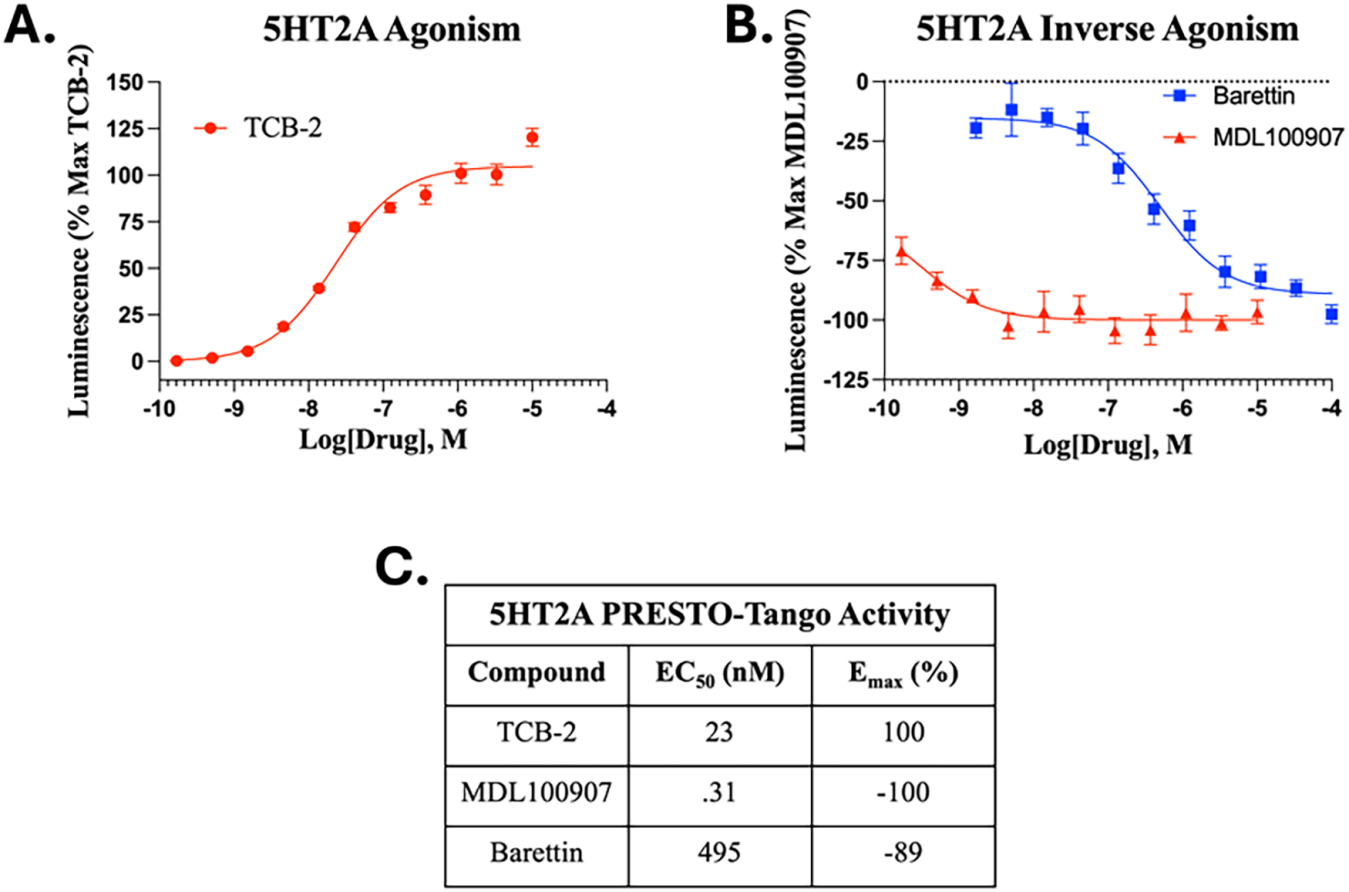
Barettin acts as an inverse agonist at the 5HT2A receptor. As described in Methods, the PRESTO-Tango assay was utilized to determine the activity of various ligands at the 5HT2A receptor. Data shown is the mean ± SEM for duplicates in 3 technical and biological replicates. Data is fitted by a nonlinear regression with 3 parameters. (A) The selective 5HT2A agonist TCB-2 activated the 5HT2A receptor. (B) Barettin produced an inverse agonist response with the known inverse agonist MDL100907 producing similar activity. (C) Activity of compounds at the 5HT2A receptor derived from nonlinear regression with 3 parameters.

This effect mirrors the activity observed with MDL100907, a well-characterized 5HT2A inverse agonist (Figure 2B), albeit barettin shows a lower potency (Figure 2C), consistent with the high doses needed to elicit the above antihyperalgesic activity.^33^ These findings support the hypothesis that barettin modulates serotonergic signaling through inverse agonism at the 5HT2A receptor, providing a potential mechanistic explanation for barettin’s antihyperalgesic effects.

### Barettin Shows No Activity at the μ-Opioid Receptor

To rule out potential confounding receptor activity of barettin, we investigated whether its antihyperalgesic effects could be mediated through activity at the μ-opioid receptor (MOR). This was investigated using the PRESTO-Tango assay to determine the activity of barettin at the MOR when compared to the highly efficacious MOR agonist DAMGO. The results of the PRESTO-Tango assay demonstrate that barettin has no activity at the MOR (Figure 3). This suggests that the antihyperalgesic mechanism of barettin is likely to be mediated through other molecular pathways and infers that barettin has the potential to act as an antihyperalgesic agent without the major adverse effects of opioids (i.e., via the 5HT2AR).

**Figure 3:**
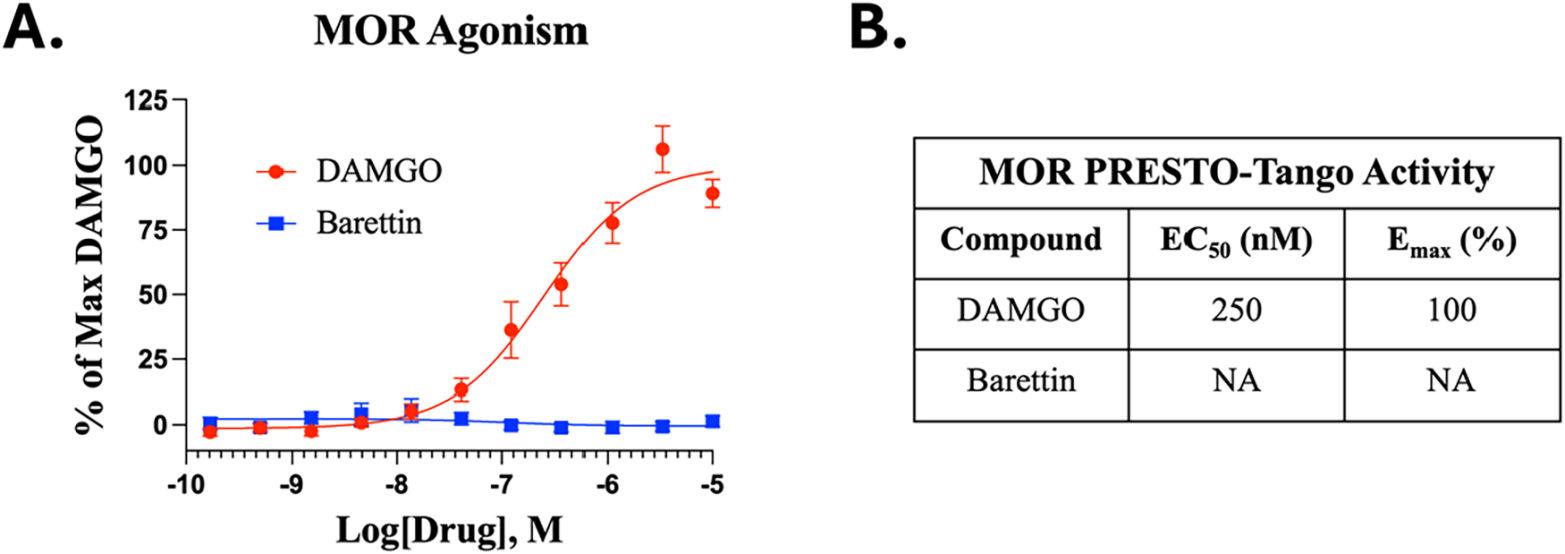
Barettin does not act as an agonist at the MOR. As described in Methods, the PRESTO-Tango assay was utilized to determine the activity of barettin at the μ-opioid receptor (MOR). Data shown is the mean ± SEM for duplicates in 3 technical and biological replicates. Data is fitted by a nonlinear regression with 3 parameters. (A) Barettin has no activity at the MOR compared to the known MOR agonist DAMGO. (B) Activity of compounds at the MOR derived from nonlinear regression with 3 parameters.

### Molecular Target Analysis: Docking at the 5HT2A

To probe further into the inverse agonism of barettin, we employed an *in silico* molecular docking approach to model barettin’s affinity for the 5HT2A receptor and to support our signaling results. Although molecular docking is constrained by the assumptions of a rigid receptor, limitations of various scoring functions, and the need for experimental validation, it provides a quick and inexpensive method to understand receptor–ligand interactions.^34^ Several structures for the human 5HT2A receptor exist in the Protein Data Bank, but to accurately model the inverse agonism of barettin, only those bound to known inverse agonists were considered. Among these, the inactive-state structure complexed with risperidone (PDB: 6A93) had the best resolution (3.00 Å) and was selected for molecular docking using the Glide-SP protocol.^35^ Barettin occupied the orthosteric site of the receptor, like risperidone, pimavanserin, and many other 5HT2A inverse agonists and antagonists, as shown in Figure 4A. At physiological pH, the guanidino group of barettin was predicted to be protonated, forming a commonly observed salt-bridge interaction with D155^3.32^ (distance 2.90 Å) seen in aminergic receptors. The conserved residue W336^6.48^ present in the bottom hydrophobic cleft (see Figure 4B), is known to act as a “toggle-switch” for receptor activation in serotonin receptors.^36^

**Figure 4:**
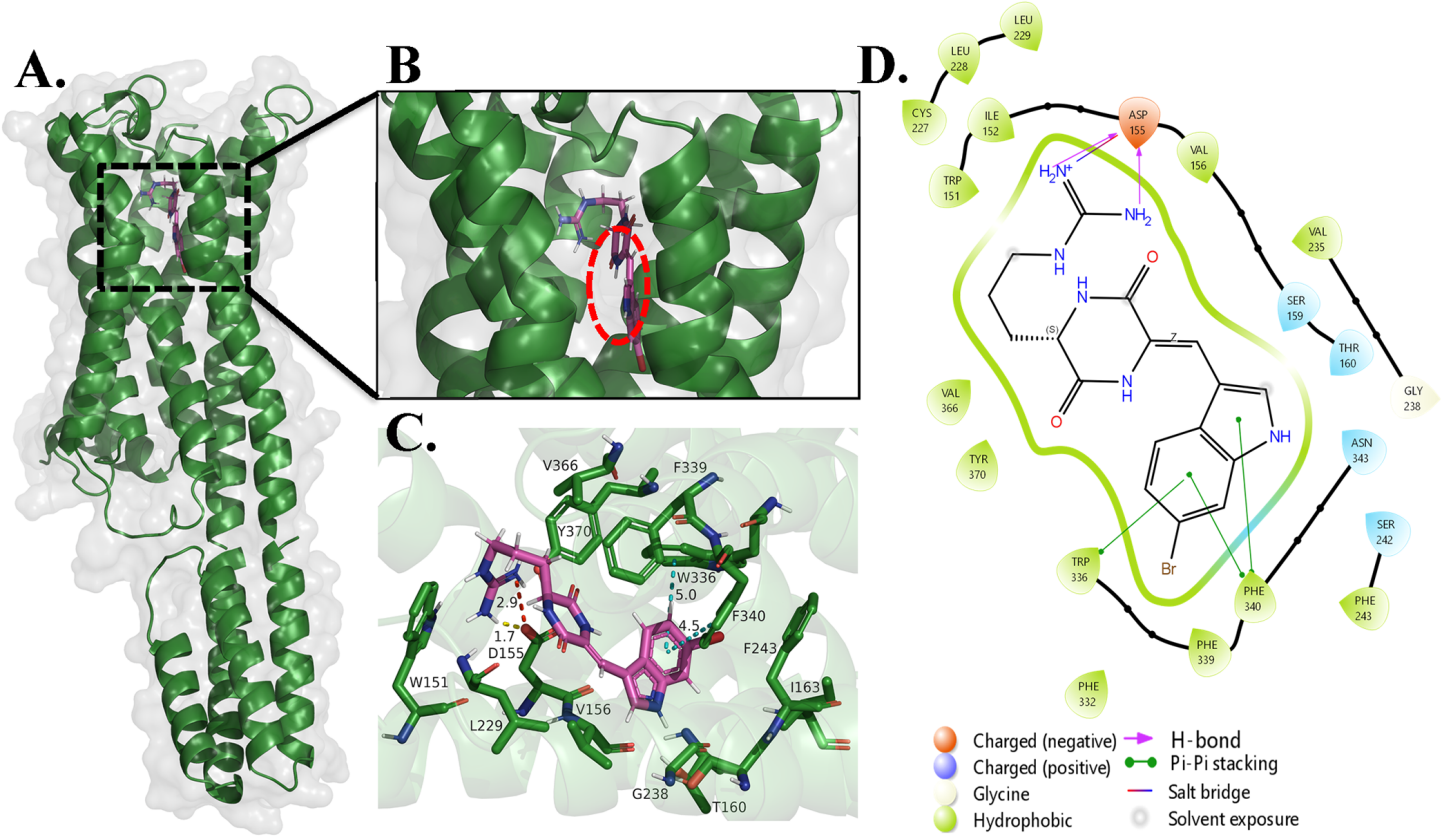
Results from molecular docking between the inactive state 5HT2A receptor (PDB: 6A93) and barettin. (A) Global view. (B) Close-up view indicating the bottom hydrophobic cleft (red-dotted circle). (C) 3D receptor–ligand interaction diagram depicting key interactions (salt-bridge – red, H-bond – yellow and π–π stacking – cyan) between barettin (magenta) and the amino acid residues in the binding site of 5HT2A (green). (D) 2D map of the interactions between 5HT2A and barettin.

The brominated indole moiety of barettin sat in this cleft and formed two edge-to-face π–π interactions with W336^6.48^ and F340^6.52^ as shown in Figure 4C. The ligand also formed other hydrophobic interactions with F243^5.43^, I163^3.40^, V366^7.39^, and F339^6.51^ (superscripts indicate Ballesteros–Weinstein numbering for conserved GPCR residues), reminiscent of those formed by other inverse agonists.^37,38^ In summary, our docking model suggests that barettin stabilizes the inactive state of the 5HT2A receptor by restricting the movement of these key residues. This further supports and expands on our molecular signaling results above.

### Confirming Antihyperalgesic Activity through Inverse Agonism of the 5HT2A

To further confirm that the antihyperalgesic effects of barettin are mediated by 5HT2A receptor inverse agonism we utilized the well characterized 5HT2A antagonist ketanserin.^39^ The effect of ketanserin on the antihyperalgesic effects of barettin was assessed *in vivo* using von Frey measurements in male CIPN mice. Notably, pretreatment with ketanserin abolished the antihyperalgesic effects of Barettin (Figure 5). Neutral antagonists such as ketanserin are expected to abolish the effects of both agonists and inverse agonists. Thus, these results combined with our *in vitro* results, strongly suggest that barettin is acting via inverse agonism of the 5HT2A receptor. To confirm these results were specific to the 5HT2a antagonist activity of ketanserin, the effect of ketanserin on the antihyperalgesic effects of the opioid agonist oxycodone was assessed (Figure 5). In contrast to barettin, pretreatment with ketanserin had no effect on the antihyperalgesic effects of oxycodone, confirming its specificity. Furthermore, ketanserin on its own has no antihyperalgesic activity (Figure 5), further confirming the specificity of its action.

**Figure 5:**
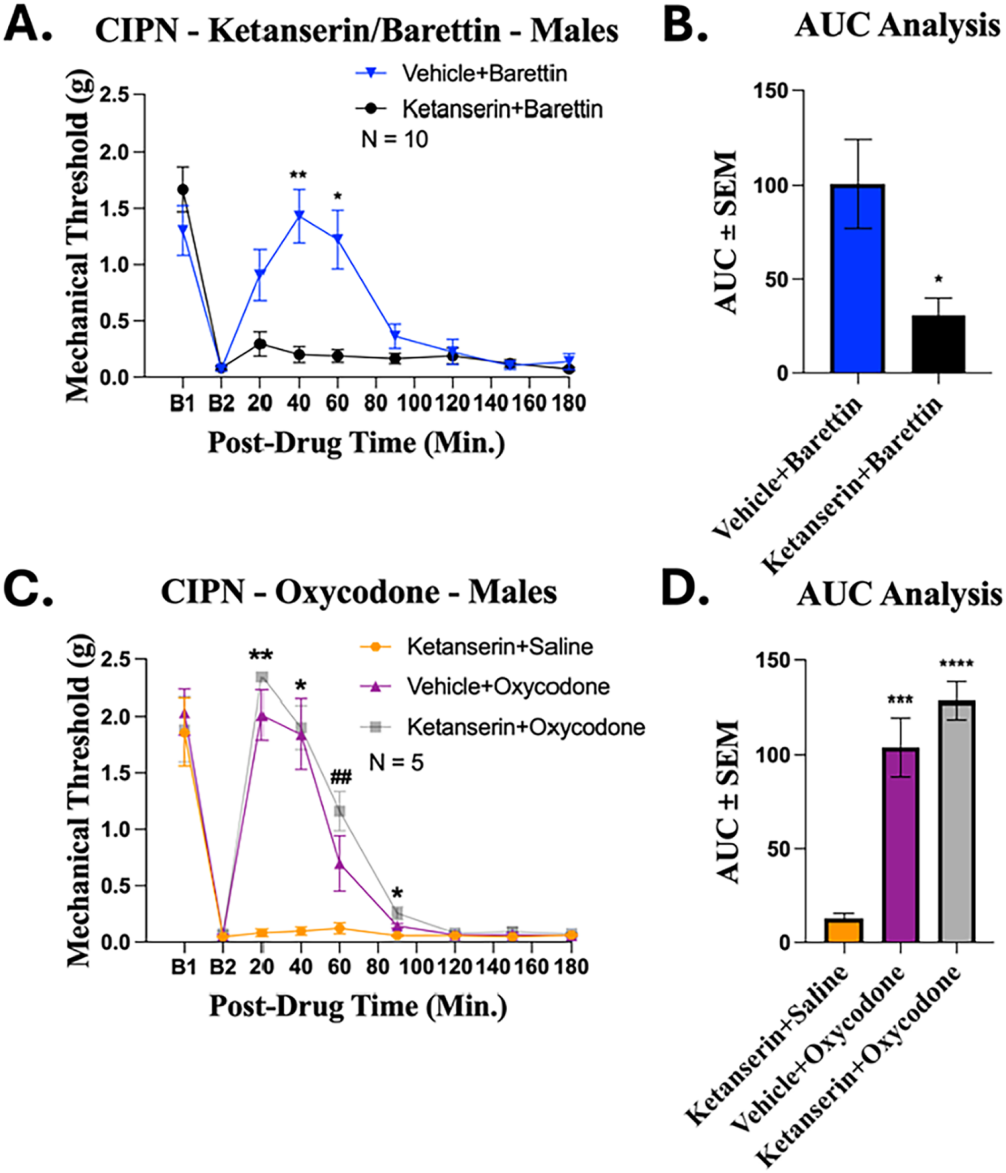
Ketanserin blocks the antihyperalgesic activity of Barettin without having any effect on the antihyperalgesic activity of oxycodone in male CIPN mice. Male CD-1 mice had CIPN induced and measured as described in the Methods. Data shown is the mean ± SEM, performed in 1–2 technical replicates for each experiment, with sample sizes noted in graph. B1 = baseline prior to CIPN induction, B2 = baseline following CIPN induction. Three mg/kg ketanserin or vehicle were injected IP, 20 min prior to IP injection of 178 mg/kg barettin (A), 5 mg/kg oxycodone, or saline (C) with mechanical allodynia measured. The Area Under the Curve (AUC) for antihyperalgesic effects were analyzed for barettin (B) and ketanserin (D), as described in Methods. (A) *, ** = *p* < 0.05, 0.01 vs same time point Ketanserin + Barettin determined by 2 way ANOVA with Šidák’s post hoc test. (B) * = *p* < 0.05 vs Vehicle + Barettin determined by two tailed Welch’s *t* test. (C) *, ** = *p* < 0.05, 0.01 Vehicle + Oxycodone or Ketanserin + Oxycodone vs same time point Ketanserin + Saline. ^##^ = *p* < 0.01 Ketanserin + Oxycodone vs same time Ketanserin + Saline, determined by 2 Way ANOVA with Tukey’s post hoc tests. (D) ***, **** = *p* < 0.001,. 0001 vs same time point Ketanserin + Saline, determined by 1 Way ANOVA with Dunnet’s post hoc test. *F* and *P* values are provided in Table S1.

Further confirmation of this mechanism was obtained via utilization of MDL-100907, a well-characterized 5HT2A inverse agonist, *in vivo* using von Frey measurement in male CIPN mice. Treatment with MDL-100907 produced significant antihyperalgesic activity, closely paralleling the effects observed with barettin (Figure 6).

**Figure 6:**
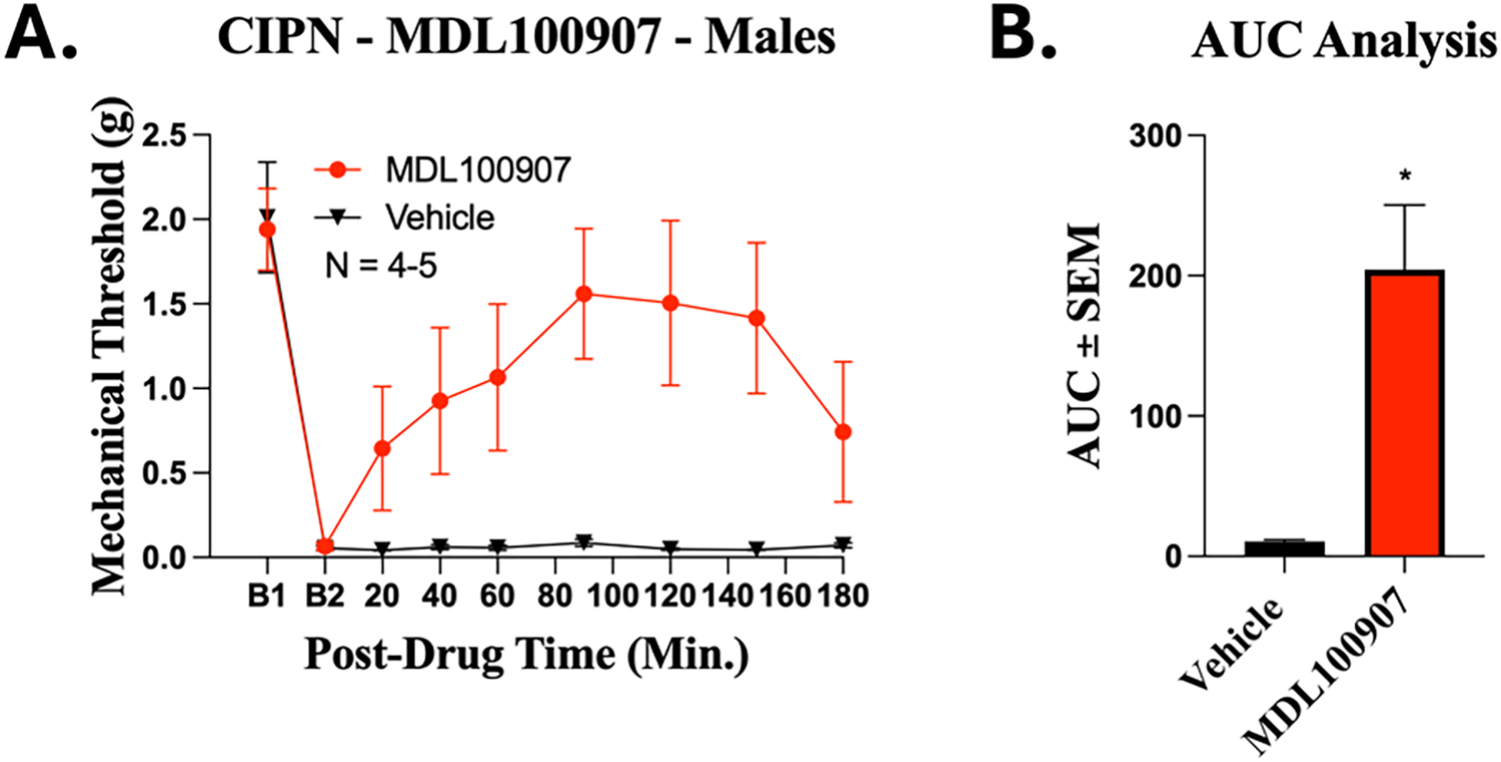
MDL100907 shows antihyperalgesic activity in mice with CIPN. Male CD-1 mice had CIPN induced and measured as described in the Methods. Data shown is the mean ± SEM, performed as 1 technical replicate, with sample sizes noted in graph. B1 = baseline prior to CIPN induction, B2 = baseline following CIPN induction. Ten mg/kg MDL100907 or Vehicle were injected IP, with mechanical allodynia measured (**A**). Two Way ANOVA with Tukey’s post hoc test performed with no significance found. The Area Under the Curve (AUC) for antihyperalgesic effects was analyzed (**B**). * = *p* < 0.05 vs Vehicle, determined by two tailed Welch’s *t* test. *F* and *P* values are provided in Table S1.

Combined, these results provide strong pharmacological evidence that inverse agonism at the 5HT2A receptor contributes to antihyperalgesic activity. This convergence of receptor pharmacology and behavioral assays strengthens the mechanistic link between barettin’s *in vitro* profile and *in vivo* analgesic effects.

### Ruling Out Hallucinogenic Properties for Barettin

Agonism of the 5HT2A receptor is strongly associated with hallucinogenic effects.^40^ As such, it was essential to rule out the hallucinogenic potential of barettin, even though inverse agonist activity would not be predicted to produce such a response. To assess this, we employed the head-twitch response (HTR) assay, a well-validated behavioral test for hallucinogenic activity mediated through 5HT2A. Male mice were administered intraperitoneal injections of 178 mg/kg barettin, 200 mg/kg 5-hydroxytryptophan (5-HTP) positive control, or vehicle, and head twitches were scored in consecutive 5 min bins for 30 min postinjection. Mice treated with 5-HTP exhibited a pronounced head-twitch response throughout the observation period. In contrast, barettin-treated mice showed no detectable head twitches, with responses indistinguishable from the vehicle group across all time bins (Figure 7). The absence of a HTR following barettin administration further supports that barettin does not act as an agonist at the 5HT2A receptor and is unlikely to elicit hallucinogenic-like effects *in vivo*.

**Figure 7:**
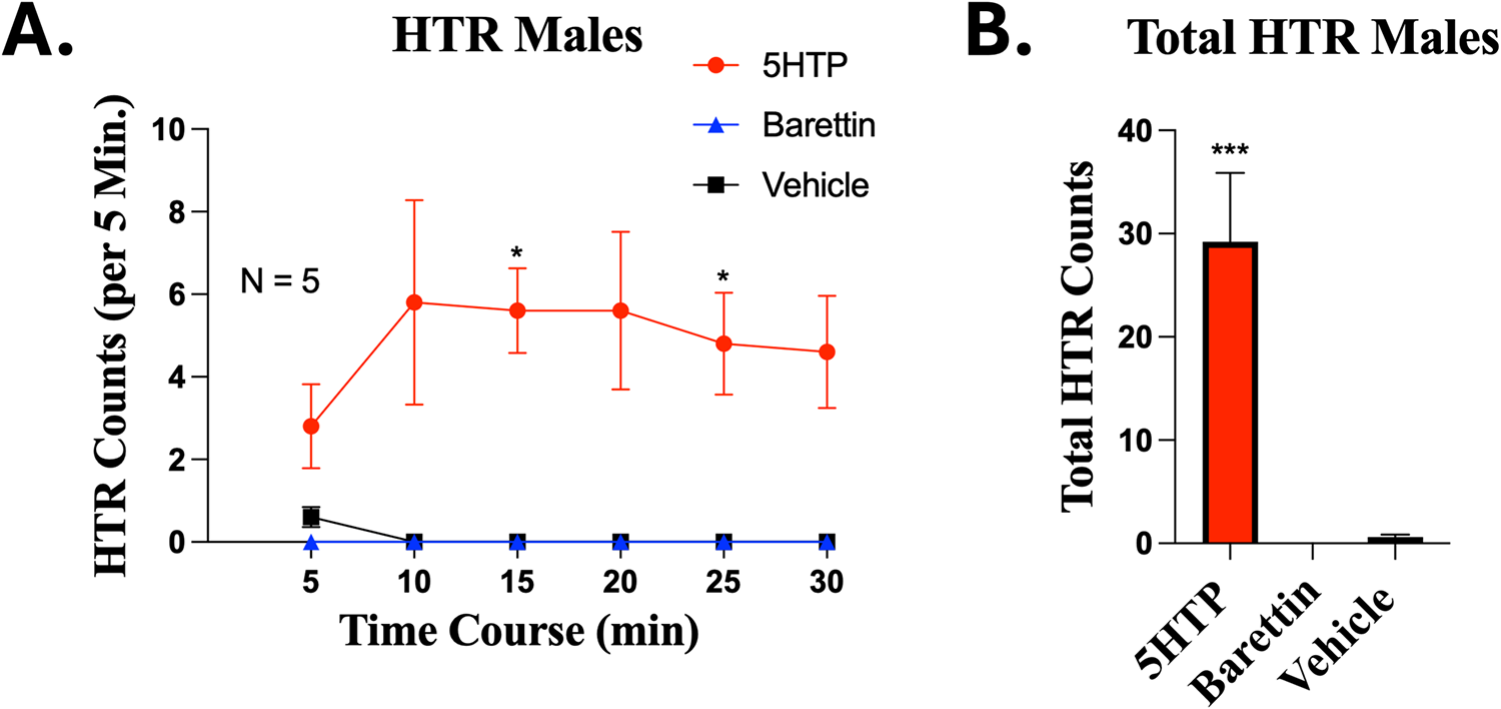
Barettin does not display hallucinogenic-like effects in the head-twitch response (HTR) assay. Male mice were administered IP injection of 200 mg/kg 5-hydroxytryptophan (5-HTP; positive control), 178 mg/kg barettin, or vehicle and HTR was measured as described in Methods. Data shown is the mean ± SEM, performed as 2 technical replicates, with sample sizes noted in graph. HTRs in 5 min bins were collected (A). * = *p* < 0.05 5HTP vs same time point Barettin or Vehicle, determined by 2 Way ANOVA with Tukey’s post hoc test. Cumulative HTRs were analyzed over the entire time course (B). *** = *p* < 0.001 vs Vehicle, determined by 1 Way ANOVA with Dunnett’s post hoc test. *F* and *P* values are provided in Table S1.

### Sex Differences in Antihyperalgesic Efficacy

The sex-specific efficacy noted in Figure 1 poses a unique translational challenge, but also a mechanistic opportunity. Existing evidence supports the occurrence of such divergence, as sex hormones are known to impact behavior at the pain circuit level, resulting in sex-specific effects.^41^ For example, evidence in preclinical studies have shown that descending pain control mechanisms which involve the rostral ventromedial medulla are more pronounced in males than females.^42,43^ Other studies have shown that 5HT2A is highly expressed within male mice dorsal root ganglia (DRG) while female mice have little to no expression.^44^ We thus hypothesize that 5HT2A inverse agonists may engage inhibitory circuits in males, whereas similar pathways may be less effectively recruited in females. It should be noted however that differences in pharmacokinetic factors such as metabolism, hormone-dependent drug transport, and blood–brain barrier permeability may also play a role in explaining lower efficacy in females. Moreover, the sex-specific neuroimmune reactions, especially regarding glial responses and cytokine signaling in response to chemotherapy-related nerve injury, may influence the effect of modulation on serotonin.^44^ These findings strongly illustrate the need to consider sex as a biological variable when assessing serotonergic analgesics like barettin, and to conduct mechanistic research to explain the observed differences in efficacy.

### Mechanistic Insights and Therapeutic Potential of 5HT2A in Pain Modulation

The 5HT2A receptor is a pivotal regulator of nociception, expressed in peripheral sensory neurons, the dorsal horn of the spinal cord,^45^ and supraspinal centers, including the periaqueductal gray (PAG) and rostral ventromedial medulla (RVM).^42^ Attempts to target the 5HT2A receptor for the treatment of pain have produced the seemingly contradictory findings that both agonism and inverse agonism are antinociceptive.^46,47^ This may be explained by 5HT2A receptors influencing pain based on the specific area and context of their activation. For example, in peripheral nociceptors 5HT2A enhances pain through Gq/11-mediated PLC–PKC signaling and increases calcium influx, whereas supraspinal 5HT2A activation promotes descending inhibition and antinociception.^48^ These findings position 5HT2A as a dynamic modulator of pain circuits, capable of shifting between pronociceptive and antinociceptive roles depending on its localization and signaling context, potentially explaining how both agonism and inverse agonism are antinociceptive.

Interestingly, in chronic pain states constitutive 5HT2A activity is linked to maladaptive excitatory tone and central sensitization.^49,50^ Unlike neutral antagonists, inverse agonists stabilize a receptor in an inactive state, thus lowering constitutive signaling, and biasing active downstream pathways to inhibitory states.^51^ The inverse agonist action of attenuating constitutive 5HT2A activity may help in managing the chronic pain state where constitutive activity is maladaptively upregulated. In the present study, barettin showed 5HT2A inverse agonism in a cell-based assay and produced antihyperalgesic effects in male mice with CIPN, supporting the idea that reduced 5HT2A signaling contributes directly to analgesia. By acting as a 5HT2A inverse agonist, barettin may counteract neuropathic hyperexcitability through both spinal and supraspinal mechanisms. Notably, our proposed model relies on barettin penetrating the blood brain barrier, which may be limited by the charged guanidine group. Thus, further pharmacokinetic studies are required to determine the accuracy of our model.

Ultimately, the efficacy profile of barettin highlights the potential of marine-derived scaffolds as templates for developing novel serotonergic analgesics while avoiding liabilities associated with opioids. However, the therapeutic window and safety profile require thorough evaluation prior to clinical use.

## Conclusions

Our results highlight barettin as a promising antihyperalgesic candidate among marine-derived natural products. Barettin produced significant antihyperalgesic activity in male mice with CIPN with no activity in female mice. Further research is required to determine the mechanism of this sexually dimorphic response. Unlike opioid compounds, barettin did not engage the MOR, confirming the nonopioid analgesic profile. Furthermore, though previous studies have identified barettin may act at 5HT2 receptors, this study is the first to identify the above *in vivo* properties and that functionally barettin acts as a 5HT2A inverse agonist. It has been shown that 5-HT2A receptor expression is increased in the spinal cord after treatment of chemotherapeutic agents. Further, the blockade of these receptors via the 5-HT2A inverse agonist MDL-100907 significantly lowered mechanical hypersensitivity. This evidence supports 5HT2A inverse agonism by barettin as the mechanism for this pain relief. The lack of head-twitch behavior response following barettin administration also supports its therapeutic profile, hinting that interactions with the serotonergic system can be exploited without the receptor-associated hallucinogenic effects that are seen with 5HT2A receptor agonists. Overall, these findings validate 5HT2A inverse agonism as a potential novel nonopioid analgesic approach and identify barettin as a lead scaffold with mechanistic and translational potential. This research further demonstrates the abundant untapped potential of marine natural products to develop unique chemical entities that address the ongoing challenges of treating chronic pain.

## Experimental Section

### Isolation of Barettin

Three specimens of *G. barretti* were collected by Karin Steffen on board the research vessel Hans Brattstrøm, with a triangular dredge, on the 8th-9th of Sept. 2016, at 250–330 m depth, in the Korsfjord and Langenuen, south of Bergen, Norway. Specimens were identified on board by sponge taxonomist Prof. H. T. Rapp, and frozen upon collection. Specimens were sent frozen to Uppsala University, Sweden, where they were freeze-dried, before being sent to the University of Arizona. The specimens used in this paper are identified with the following museum accession numbers Uppsala Zoological Museum Collection UPSZMC 195453, UPSZMC 195454, and UPSZMC 195455. Collection of these sponges were completed within the framework of the SponGES project (grant agreement No. 679849), funded by the European Union’s Horizon 2020 research and innovation program. The collections were done by the University of Bergen (Norway), a partner of the SponGES project. Freeze-dried sponge extract was placed on filter paper in a funnel and rinsed with dichloromethane (DCM) to wash away lipid contaminants in the sample.^52,53^ After the sample was rinsed with DCM, the freeze-dried extract was washed with a solution of 60% acetonitrile. These washes were analyzed using LC-MS and were later combined. The sample was concentrated to a final volume of 1.5 mL. The barettin was then purified using RP-HPLC on a Phenomenex Luna C18 (5-μm, 250 × 21.2 mm) column with UV detection at 234 nm. The compound was separated using a linear gradient, beginning with 95% solvent A (0.1% formic acid in Milli-Q water) and 5% solvent B (acetonitrile) and over 40 min advancing to 40% solvent B. Then solvent B was increased to 95% over 15 min, held for 5 min, and returned to the starting conditions. The retention time for barettin was determined to be 37 min with structure confirmed by NMR Spectroscopy (Supporting Figures S1 and S2) and LC-MS analysis (Figure S3).^27,28^

### Drug Preparation

All solutions were made fresh for each experiment and used immediately. Matched vehicle injections were included in each experiment as a control. Barettin, ketanserin, and MDL100907 were dissolved in 10% DMSO, 10% Tween 80, and 80% USP saline for injections. 5-Hydroxytryptophan was dissolved in 10% DMSO and 90% USP saline for injections. Oxycodone was dissolved in USP saline for injections. Paclitaxel was dissolved in 15:15:70 Cremophor-EL:ethanol:USP saline for injection. For in vitro experiments, 100 mM stock solutions of TCB-2, MDL100907, and barettin and 10 mM stock solutions of DAMGO in DMSO were diluted to the required concentration using DMEM. Final DMSO concentration was set at 0.5% in all wells. Vehicle wells with 0.5% DMSO were utilized as a control.

### Animals

Male and female CD-1 mice, ranging in age from 5 to 8 weeks, were obtained from Charles River Laboratories (Wilmington, MA) and were used in all experiments. The mice underwent a period of acclimatization in the temperature and humidity-controlled vivarium at the University of Arizona for a minimum of 5 days following their arrival. A 12-h light-dark cycle (7 AM to 7 PM) was established, with *ad libitum* access to standard chow and water. Animals were allowed to acclimate to the experimental room for a period of 30 min prior to commencing any procedures. Each experiment utilized naïve mice, and no mice were subjected to multiple tests or were reused. Animals were randomized and block assigned to treatment group by cage. The experimenters were blinded to treatment by the delivery of coded drug vials; unblinding only occurred after the collection of all data. All experiments conducted at the University of Arizona were approved by the Institutional Animal Care and Use Committee (IACUC). Experiments were conducted in compliance with the NIH Guide for the Care and Use of Laboratory Animals and the International Association for the Study of Pain Guidelines for the Use of Animals in Research.

### Von Frey Mechanical Threshold Measurement

Mice were placed in a clear chamber with a wire mesh floor and allowed to acclimate for a minimum of 30 min prior to assay.^54^ Von Frey Filaments with different force thresholds (2.44, 2.83, 3.22, 3.61, 4.08, 4.31, and 4.56 g) were applied to the hind left paw of the mouse and foot recoil was recorded. Mice were tested prior to any procedures and recorded as their baseline threshold. The up–down paradigm was used to determine mechanical thresholds with four measurements taken after the first response per mouse.^14^

### Chemotherapy Induced Peripheral Neuropathy

To induce CIPN, mice received intraperitoneal injections of 2 mg/kg paclitaxel on days 1, 3, 5, and 7 to elicit neuropathy. Baseline mechanical sensitivity was measured prior to the administration of paclitaxel on day 1. Neuropathy was measured on day 8, after which barettin, MDL100907, or vehicle was administered via intraperitoneal injection. Mechanical sensitivity was assessed over a 3 h time course following injection using von Frey filaments as above.^14^

### Head Twitch Response

The hallucinogenic effects of barettin were assessed using the head twitch response (HTR) assay. Male mice were allowed to acclimate in a clear cylinder for a minimum of 30 min prior to the experiment. After acclimation, intraperitoneal injections of 5-HTP (200 mg/kg), barettin (178 mg/kg), or vehicle were administered. Mice were then recorded for 30 min postinjection. These videos were anonymized and given to a separate researcher to analyze for the presence of head twitches. Time where head twitches occurred and the total number of head twitches were recorded. The twitches were combined into 5 min bins for analysis.^55^

### Cell Culture

HTLA cells were cultured in DMEM supplemented with 10% dialyzed FBS (Fisher Scientific, #A3382001) or FBS (ThermoFisher, #s10437028) for 5HT2A and MOR experiments respectively, 1% Penicillin/Streptomycin, 100 μg/mL hygromycin B, and 2 μg/mL puromycin. Cells were grown in a 5% CO_2_ atmosphere at 37 °C. Experiments were performed with cells from passages 5 to 20.

### PRESTO-Tango Assay

The PRESTO-Tango assay is a sensitive, cell-based technique specifically developed to track GPCR activation through β-arrestin recruitment, one of the most common downstream responses to the activation of any GPCR. Specificity of this assay is achieved by the necessary interaction between both a modified β-arrestin and a modified GPCR. The PRESTO-Tango assay provides integrated measurements for activity of receptors and thus can accurately differentiate between agonist, antagonist and inverse agonist activities. HTLA cells were used for the PRESTO-Tango Assay as in previous works and cultured as above.^56^ Each 1/3 of a confluent plate was suspended in 500 μL of 1.07 mg/mL BES in DMEM. 500 μL of cells and 10 μg of 5HT2A receptor (Addgene, #66409) or MOR (Addgene, #66464) PRESTO-Tango DNA constructs were added to a 4 mm electroporation cuvette. Electroporation was performed in one pulse using an exponential protocol with Voltage = 260 V and Capacitance = 1000 μF. After transfection, 100 K cells per well were plated in poly-l-lysine coated 96 well plates (white wall, clear bottom) and allowed to recover overnight. For the MOR, cells were then serum-starved for 4 h followed by treatment with vehicle or test compounds. For 5HT2A, no serum-starve was performed and test compounds were immediately applied. After overnight incubation at 37 °C, the cells were treated with a 1:20 dilution of Bright-Glo (Promega) in Tango assay buffer (HBSS with 20 mM HEPES).^57^ After a 20 min incubation at room temperature, luminescence was measured without filters using a CLARIOstar Plus plate reader, with top-read and 5 s acquisition time.

### Molecular Docking

Molecular docking was performed using Schrödinger Maestro Release 2025-3. The receptor structure was procured from the Protein Data Bank (PDB: 6A93) and prepared for docking using the Protein Preparation Workflow (default parameters) available in Schrödinger Maestro. Three-dimensional coordinates for the ligand barettin were obtained using LigPrep, and the Receptor Grid Generation tool was used to generate a 20 × 20 × 20 Å grid centered on the active site of the receptor. Barettin was then docked using the Glide-SP protocol with default parameters, asking for up to 10 poses.^35^ The docked poses were exported as a PDB file and PyMOL 3.1.6.1 was used for visualizing the data and generating images.

### Data Analysis

All data were reported as the mean ± SEM. The behavioral data were reported raw, without Maximum Possible Effect or other normalization. Males and females were not combined due to noted sex differences. Area Under the Curve (AUC) was calculated for time course data sets; this was done using the AUC function with the prepain baseline data point excluded from analysis. For Figures 1, 5, and 6, time course analysis was performed by 2 way ANOVA with Tukey’s post hoc test and AUC analysis was performed by 1 Way ANOVA with Dunnet’ post hoc test or Šidák’s post hoc test (Figure 5B). For Figure 7, time course analysis was performed by 2 way ANOVA with Tukey’s post hoc test and total HTRs was analyzed using 1 Way ANOVA with Dunnett’s post hoc test. All 2 way ANOVA used the Geisser-Greenhouse correction to account for a potential lack of sphericity of the data. For Figure 1A, since males showed significant differences between the doses, a dose/response curve was constructed. This was accomplished by defining the mean pre-CIPN and post-CIPN baselines as the Maximum Possible Effect (MPE) window and transforming the peak response time measurements for each group (e.g., 60 min) to fit within this window (0–100%). The peak dose effect was then graphed and a linear regression fitted to the dose curve. The curve was further interpolated at the 50% mark to calculate the ED50. For PRESTO-Tango, the average luminescence in the vehicle wells (0.5% DMSO) was subtracted from all wells to set baseline signaling. Wells were then analyzed as percent of maximum effect for the given control, determined by nonlinear regression with 3 parameters. PRESTO-Tango data is represented as normalized data points and associated nonlinear regression with 3 parameters. All graphing and statistical analysis was performed using GraphPad Prism 10.4.2. All *F* and *P* values are provided in Table S1.

## Supplementary Material



## Data Availability

All data required to interpret these findings are contained within the manuscript or Supporting data. NMR data ^1^H and ^13^C can be viewed using the NP-MRD,^58^ NP-CARD ID: NP0068647 with MS data available upon request to the corresponding author.

## Notes

^1^H, ^13^C NMR, and HRMS (PDF) spectroscopic data (Figures S1–S3) and statistical values (Table S1) (PDF)

## ◆

C.A.S and M.O. to be considered as joint authors on this publication.
