# Supplementary material for: Barettin, a Nonopioid, Nonhallucinogenic Marine Natural Product with Antihyperalgesic Properties Mediated by 5HT2A Inverse Agonism
Source: J Nat Prod. 2026 Mar 31;89(4):1298–308. doi: 10.1021/acs.jnatprod.6c00169 (PMC13122638; doi:10.1021/acs.jnatprod.6c00169)
Supplement: Supplementary file 1 [file np6c00169_si_001.pdf]

Supporting Information.

Barettin, a Non-Opioid, Non-Hallucinogenic Marine Natural Product with Antihyperalgesic Properties Mediated by 5HT2A Inverse Agonism.

Caleb A. Seekins[1†], Michael Okine[2b†], Emily G. Forrest[1], Timothy Chavez[2b], Alexandra Stump[1], Vishal Kaleeswaran[2a], Jerry E. Carr[1], Christopher Hulme[2], Paco Cardenas[3,4], Todd W. Vanderah[1,5], John M. Streicher[1,5]* and Christopher Cartmell[1,5,6]*.

[1] Department of Pharmacology, College of Medicine- University of Arizona, Tucson, Arizona, USA, 85724

[2a] Department of Pharmacology & Toxicology, College of Pharmacy, and [2b]Department of Chemistry and Biochemistry, College of Science - University of Arizona, Tucson, Arizona, USA. 85724

[3] Pharmacognosy, Department of Medicinal Chemistry - Uppsala University, Sweden, 75337

[4] Museum of Evolution - Uppsala University, Sweden,75237

[5] Comprehensive Center for Pain and Addiction - University of Arizona, Tucson, Arizona, USA 85724

[6] University of Arizona Cancer Center - University of Arizona, Tucson, Arizona, 85724 and the Center for Applied Nano Bioscience and Medicine, College of Medicine - University of Arizona, Phoenix, Arizona, USA, 85004

† To be considered joint authors on this publication

* Correspondence: jstreicher@arizona.edu and cartmell@arizona.edu

^{1}H NMR of Barettin (Figure S1) 2

^{13}C NMR of Barettin (Figure S2) 3

LC and HRMS traces (Figure S3) 4

Statistical Details for Main Figure (Table S1) 5

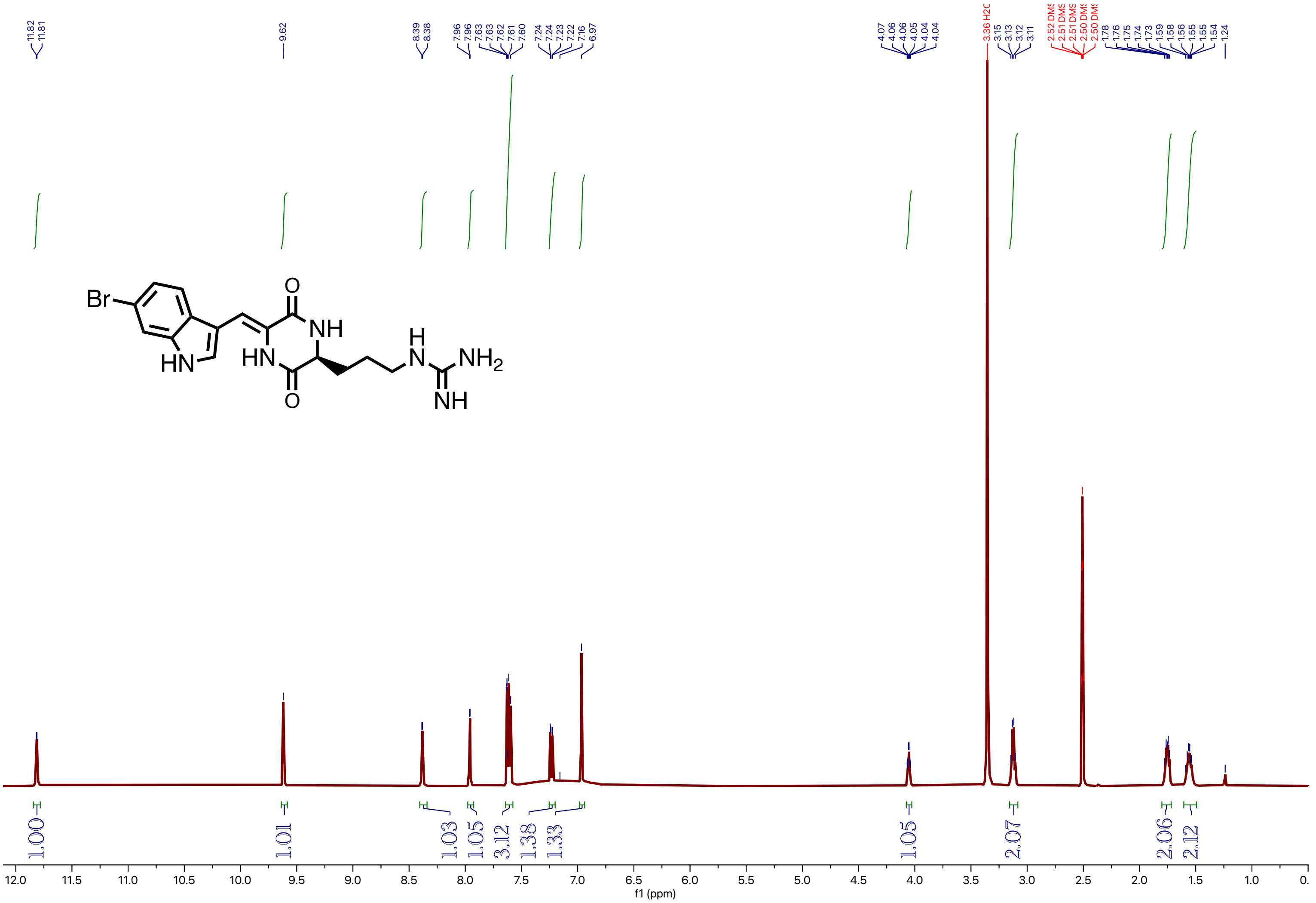

Figure S1: ^{1}H NMR of Barettin

^{1}H NMR (500 MHz, DMSO) δ 11.82 (s, 1H), 9.65 (s, 1H), 8.40 (d, *J* = 2.7 Hz, 1H), 7.96 (d, *J* = 2.7 Hz, 1H), 7.65 – 7.56 (m, 3H), 7.23 (d, *J* = 8.5 Hz, 1H), 6.96 (s, 1H), 4.05 (td, *J* = 5.7, 2.6 Hz, 1H), 3.12 (q, *J* = 6.7 Hz, 2H), 1.74 (dd, *J* = 9.5, 5.6 Hz, 2H), 1.55 (h, *J* = 6.5 Hz, 2H).

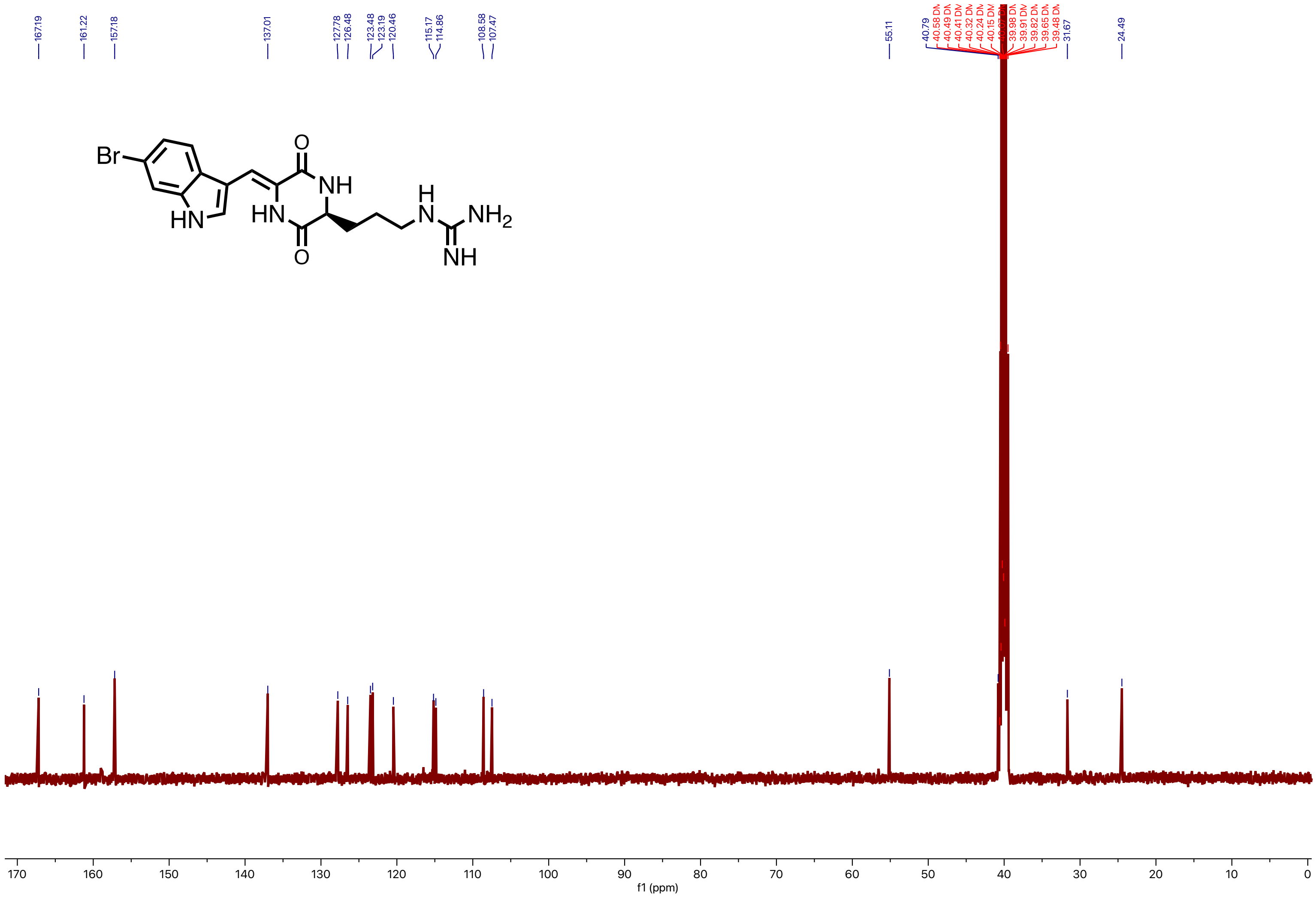

Figure S2: ^{13}C NMR of Barettin.

^{13}C NMR (126 MHz, DMSO) δ 167.2 (CO), 161.2 (CO), 157.2 (C(NH)$_2$NH$_2$,) 137.0 (C), 127.8 (CH), 126.5 (C), 123.5 (CH), 123.2 (CH), 120.5 (CH), 115.2 (C), 114.9 (CBr), 108.6 (CH), 107.5 (C), 55.1 (CH), 40.5 (CH$_2$), 31.7 (CH2), 24.5 (CH$_2$).

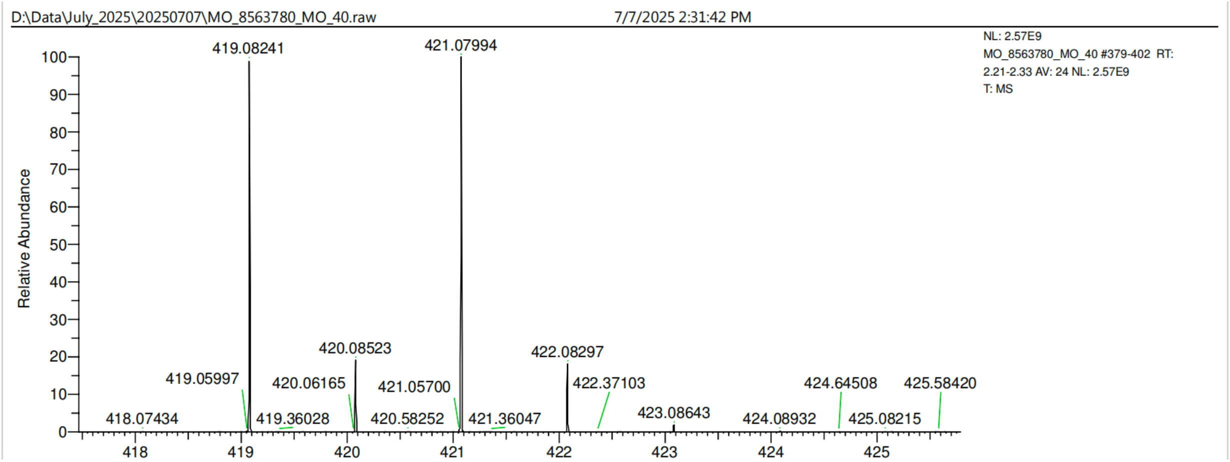

***Figure S3*:** LC and HRMS traces

MS (ESI) *m/z*, 419 (100) [M (^{79}Br) +H]$^+$, 421 (100) [M(^{81}Br) + H]$^+$; **HRMS (FTMS + p ESI):** m/z calculated for: $C_{17}H_{20}BrN_6O$ [M (^{79}Br) + H]$^+$: 419.0826; found: 419.0824.

Table S1: Statistical details for the main text. ANOVA F values with degrees of freedom (DFn,DFd) reported for each ANOVA comparison in the main text

Statistical Details								
Group	Subject		Treatment		Time		Interaction	
	F Values	P Values	F Values	P Values	F Values	P Values	F Values	P Values
Figure 1A	F (34, 272) = 4.263	P<0.0001	F (3, 34) = 9.588	P<0.0001	F (3.982, 135.4) = 48.17	P<0.0001	F (11.95, 135.4) = 3.207	P=0.0005
Figure 1B			F (3, 34) = 7.394	P=0.0006				
Figure 1C	F (19, 152) = 2.207	P=0.0044	F(3,19) = 0.1543	P=0.9256	F (1.620, 30.77) = 75.07	P<0.0001	F (4.859, 30.77) = 0.3302	P=0.8865
Figure 1D			F (3, 19) = 0.2297	P=0.8746				
Figure 5A	F (18, 144) = 2.644	P=0.0007	F (1, 18) = 10.64	P=0.0043	F (3.326, 59.86) = 28.64	P<0.0001	F (3.326, 59.86) = 9.313	P<0.0001
Figure 5B				P=0.018				
Figure 5C	F (12, 96) = 0.8811	P=0.5685	F (2, 12) = 57.53	P<0.0001	F (2.744, 32.93) = 87.21	P<0.0001	F (5.489, 32.93) = 12.68	P<0.0001
Figure 5D			F (2, 12) = 31.74	P<0.0001				
Figure 6A	F (7, 56) = 4.212	P=0.0009	F (1, 7) = 9.812	P=0.0166	F (3.171, 22.20) = 7.955	P=0.0008	F (3.171, 22.20) = 2.292	P=0.1031
Figure 6B				P=0.0135				
Figure 7A	F (12, 60) = 4.750	P<0.0001	F (2, 12) = 18.61	P=0.0002	F (1.278, 15.34) = 0.5222	P=0.5242	F (2.557, 15.34) = 0.9945	P=0.4106
Figure 7B			F (2, 12) = 18.61	P=0.0002				
